# Advances in nitrogen-containing helicenes: synthesis, chiroptical properties, and optoelectronic applications

**DOI:** 10.3762/bjoc.21.106

**Published:** 2025-07-11

**Authors:** Meng Qiu, Jing Du, Nai-Te Yao, Xin-Yue Wang, Han-Yuan Gong

**Affiliations:** 1 College of Chemistry, Beijing Normal University, Xinjiekouwaidajie 19, Beijing, 100875, China

**Keywords:** azahelicene, chiroptical properties, circularly polarized luminescence (CPL), heteroatom containing, optoelectronic applications

## Abstract

Helicenes, a class of non-planar polycyclic aromatic hydrocarbons composed of *ortho*-fused aromatic rings forming helical architectures, have attracted considerable attention due to their intrinsic chirality and tunable optoelectronic properties. Among them, nitrogen-doped helicenes (azahelicenes) and their heteroatom-co-doped counterparts – such as B/N-, O/N-, S/N-, and Se/N-doped helicenes – have emerged as highly versatile scaffolds for chiral optoelectronic applications. The incorporation of nitrogen enables precise modulation of electronic structures, redox characteristics, and intermolecular interactions, thereby enhancing performance in circularly polarized luminescence (CPL), thermally activated delayed fluorescence (TADF), and chiral sensing. Notably, recent developments have yielded π-extended, structurally robust, and stimuli-responsive azahelicenes exhibiting record-high dissymmetry factors (|*g*_abs_| and |*g*_lum_|), elevated CPL brightness (*B*_CPL_), and efficient integration into CPL-OLEDs and redox-switchable emitters. Boron–nitrogen co-doping strategies, in particular, have facilitated the development of materials with ultra-narrowband emissions, near-unity photoluminescence quantum yields, and electroluminescence dissymmetry factors (|*g*_EL_|) exceeding 10^−3^. Likewise, heteroatom co-doping with oxygen, sulfur, or selenium enables spectral tuning across the visible to near-infrared range, improved photostability, and dual-state emissive behavior. In parallel, significant progress in synthetic methodologies – including enantioselective catalysis, electrochemical cyclizations, and multicomponent reaction systems – has granted access to increasingly complex helicene frameworks with well-defined chirality. This review systematically summarizes recent advancements in the synthesis, structural engineering, and chiroptical performance of nitrogen-doped helicenes and their heteroatom-doped derivatives, emphasizing their potential as next-generation chiral optoelectronic materials and outlining future directions toward multifunctional integration and quantum technological applications.

## Introduction

Helicenes, a class of non-planar polycyclic aromatic hydrocarbons characterized by *ortho*-fused aromatic rings forming a helical framework, have attracted significant attention due to their inherent chirality, unique optoelectronic properties, and wide-ranging applications in asymmetric catalysis [1–2], molecular recognition [3], and organic electronics [4–5]. In recent years, the incorporation of heteroatoms – particularly nitrogen – into the helicene backbone, giving rise to so-called "azahelicenes", has emerged as a powerful strategy to modulate electronic structures, enhance solubility, and expand functional diversity [6]. Substituting carbon atoms with electron-deficient nitrogen atoms introduces new opportunities to fine-tune redox potentials, charge-transport behavior, and intermolecular interactions [7]. These modifications have proven especially valuable in applications such as organic light-emitting diodes (OLEDs) [8], circularly polarized luminescence (CPL) [9], and chiral photocatalysis [10]. In the past decade, heteroatom-containing helicenes have attracted increasing attention due to their tunable optoelectronic properties and potential applications in chiral optoelectronics. Several comprehensive reviews have examined specific classes of these molecules. Crassous and co-workers provided a detailed overview of heterohelicenes up to 2019, focusing on their structural diversity and functional applications [11]. Nowak-Król and colleagues reviewed boron-doped helicenes, emphasizing their roles in chiral materials design [12], while Maeda and Ema explored the circularly polarized luminescence (CPL) properties of azahelicenes [13]. However, despite these valuable contributions, a dedicated and up-to-date overview of nitrogen-doped helicenes – particularly those incorporating additional heteroatoms within the helical π-conjugated framework – remains lacking.

This review addresses this gap by systematically summarizing recent advances (from the past five years) in the synthesis, structural modification, and chiroptical properties of nitrogen-doped helicenes. Particular attention is given to multi-heteroatom systems co-doped with elements such as boron, oxygen, sulfur, and selenium, highlighting their influence on CPL performance and structure–property relationships. We classify the nitrogen-doped helicenes into only N-containing helicenes, B,N-containing helicenes, and X,N-containing helicenes (X = O, S or Se). In each section, structurally similar compounds are categorized into groups to facilitate comparison. Then, the others are discussed in chronological order based on their reported publication dates, with attribution to the respective research groups. Notably, helicenes bearing nitrogen atoms located outside the conjugated system are excluded from this discussion to maintain a consistent focus on electronically integrated heteroatom-doped architectures.

## Review

### N-Containing helicenes

Among nitrogen-containing helicenes, HBC-fused azahelicenes represent a particularly significant subclass due to their extended π-conjugation and potential for enhanced chiroptical properties. Over the past few years, multiple research groups have investigated their synthesis, structural characteristics, and optoelectronic behavior. Notably, in 2021, Jux and co-workers reported a series of superhelicenes that combine helical and planar π-systems. However, the structural characterization of compound **1** (Table 1) was impeded by its inherent instability, limiting further analysis [14]. In 2024, Liu’s group developed a series of nonalternant nanographenes **2a**–**c** featuring a nitrogen-embedded cyclopenta[*ef*]heptalene core [15]. These compounds exhibit λ_abs_ at 363, 452, and 580 nm, and PLQYs of 0.05, 0.33, and 0.32, respectively. While compounds **2a** and **2b** display broad emission near 505 nm, **2c** shows dual-emission peaks at 588 and 634 nm with an ultranarrow FWHM of 22 nm. Notably, **2b** and **2c** demonstrate strong chiroptical activity with |*g*_abs_| values of 6.7 × 10^−3^ and 1.0 × 10^−2^, |*g*_lum_| of 2.4 × 10^−3^ and 7.0 × 10^−3^, and *B*_CPL_ values of 9.1 and 95.2 M^−1^ cm^−1^, respectively. Shortly thereafter, Gong’s group further expanded the π-system by constructing a tris-hexabenzo[7]helicene **3** with a carbazole core, which emits at 595/628 nm (PLQY = 0.40), displays |*g*_abs_| = 2.98 × 10^−3^, and achieves a *B*_CPL_ of 32.5 M^−1^ cm^−1^ [16]. In 2025, Babu’s group synthesized two regioisomeric π-extended azahelicenes, **4a** and **4b**, which differ in the position of attachment to the carbazole core [17]. Compared to **4a**, compound **4b** exhibits bathochromic shifts of 12 nm in absorption and 45 nm in emission, as well as a higher Φ_F_ (0.75 vs 0.68). Both isomers display TADF at room temperature and phosphorescence at 77 K. Notably, **4a** demonstrates a long-lived red afterglow persisting for up to 30 seconds. In contrast, **4b** exhibits superior chiroptical properties, with |*g*_abs_| and |*g*_lum_| values of 3.91 × 10^−3^ and 1.12 × 10^−3^, respectively, and an impressive *B*_CPL_ of 45.77 M^−1^ cm^−1^ (Table 1).

**Table 1:** Structures and optical properties of compounds **1**, **2a**–**c**, **3**, and **4a**,**b**.^a^

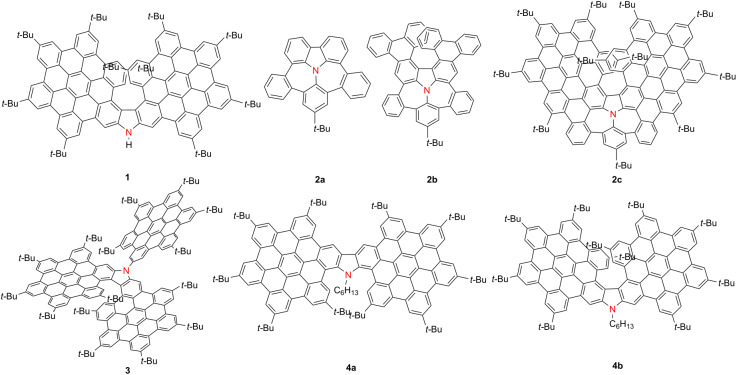						

compound	λ_abs(max)_ [nm]	λ_em_ [nm]	Ф_F_	*|g*_abs_ *|*	|*g*_lum_|	*B*_CPL_ [M^−1^ cm^−1^]

**2a**	363	508	0.05	–	–	–
**2b**	452	503	0.33	6.7 × 10^−3^	2.4 × 10^−3^	9.1
**2c**	580	588, 634	0.32	1.0 × 10^−2^	7.0 × 10^−3^	95.2
**3**	525	595, 628	0.40	2.98 × 10^−3^	4.3 × 10^−4^	32.5
**4a**	497	497, 531, 570	0.677	–	–	–
**4b**	522	542, 581, 630	0.754	3.91 × 10^−3^	1.12 × 10^−3^	45.77

^a^Compound **1** is unstable and characterized only by mass spectrometry.

In 2021, several research groups reported structurally diverse heterohelicene systems exhibiting distinctive chiroptical and photophysical properties, highlighting the expanding potential of these molecules in chiral optoelectronics. Yorimitsu’s group developed a series of dihetero[8]helicenes through a systematic asymmetric synthesis. Among these, diaza[8]helicene **5** exhibited pronounced chiroptical activity, with absorption and emission maxima (λ_abs_ = 399 nm, λ_em_ = 405 nm), a fluorescence quantum yield (Φ_F_) of 0.13, and high dissymmetry factors (|*g*_abs_| = 1.9 × 10^−2^, |*g*_abs_| = 9.5 × 10^−3^ at 403 nm) [18] (Table 2). Miura and co-workers employed Pd(II)/Ag(I)-catalyzed cyclizations to construct azahelicenes, with compound **6** exhibiting enhanced chiroptical performance and protonation-induced CPL amplification [19]. Meanwhile, Audisio’s team developed heterohelicenes via regioselective [3 + 2]-cycloadditions, with compound **7** displaying pH-responsive CPL sign inversion (|*g*_lum_| = +1.1 × 10^−3^ at 430 nm, −1.2 × 10^−3^ at 585 nm) attributed to reversible intramolecular charge transfer [20]. In parallel, several groups explored the functional versatility of heterohelicenes in device-oriented and sensing applications. Crassous’s group synthesized bipyridine-embedded helicenes via the Mallory reaction, enabling coordination with Ru(II) to form NIR-emissive complexes that exhibit redox-responsive chiroptical switching, notably with complex **8** showing reversible electronic circular dichroism (ECD) upon oxidation [21]. Liao and co-workers introduced a narrowband CP-TADF emitter **9**, characterized by a narrow emission bandwidth (FWHM = 36 nm), |*g*_lum_| = 1.1 × 10^−3^, |*g*_EL_| = 1.5 × 10^−3^, and an external quantum efficiency (EQE) of 0.14 – demonstrating promise for CPL-OLED applications [22]. Wanichacheva’s team reported urazole-functionalized aza[5]helicene **10**, exhibiting selective Fe(III) sensing, marked solvatochromism, and a large Stokes shift (85 nm) with emission at 530 nm in DMSO [23] (Table 2). Collectively, these studies underscore the structural versatility and functional tunability of heterohelicenes, establishing them as robust platforms for advanced chiral optoelectronic materials. Their diverse response to external stimuli, modular synthetic accessibility, and strong CPL performance render them ideal candidates for applications in molecular sensing, stimuli-responsive switches, and next-generation CPL-active devices.

**Table 2:** Structures and optical properties of compounds **5**–**10**.

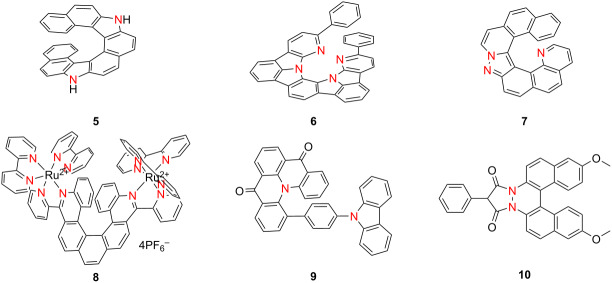						

compound		λ_abs(max)_ [nm]	λ_em_ [nm]	Ф_F_	*|g*_abs_ *|*	|*g*_lum_|

**5**		399	405, 430, 460	0.13	1.9 × 10^−2^	9.5 × 10^−3^
**6**		405	420, 439	0.14	1.1 × 10^−2^	4.4 × 10^−3^
**7**		430	436, 460, 500	0.10	–	1.1 × 10^−3^
**8**	(*M,Λ,Λ*)	522	788	0.10	–	–
	(*P,Λ,Λ*)	512	786	0.25	–	–
**9**		440	467	0.47^a^	–	1.1 × 10^−3^
**10**		400	485	–	–	–

^a^As detected in film.

In 2021, Ema’s group reported the synthesis of carbazole-based azahelicenes **11a**–**e** via intramolecular Scholl reactions [24] (Table 3). All compounds exhibited strong absorption in the UV–vis region (250–450 nm) and fluorescence emission between 400–550 nm. Among these, compound **11c**, a saddle-shaped dibenzodiaza[8]circulene, was particularly noteworthy as the first example of its kind synthesized in solution and structurally confirmed via single-crystal X-ray diffraction. It demonstrated the highest CPL performance among the series, with a |*g*_lum_| value of 3.5 × 10^−3^ and a photoluminescence quantum yield (PLQY) of 0.31, indicating its potential as a chiral emissive material. Building upon this foundation, the same group in 2024 developed a series of structurally refined aza[7]helicenes (compounds **12a** and **12b**) under modified Scholl reaction conditions [25]. These products were obtained as optically active diastereomers, which were successfully separated using silica gel chromatography. Additionally, two cyclic dimers, designated as compounds **12c** and **12d**, were isolated, exhibiting strong absorption bands at 493 and 474 nm, high PLQYs of 0.61 and 0.54, and notable CPL activity (|*g*_lum_| = 0.74 × 10^−3^ and 1.3 × 10^−3^, respectively), with corresponding brightness values (*B*_CPL_) reaching 19 and 31 M^−1^ cm^−1^ (Table 3). Importantly, both dimers displayed selective fluoride ion recognition through hydrogen bonding, with (*M*,*M*)-**12c** exhibiting a high binding constant (*K*_a_ = 2 × 10^5^ M^−1^). The resulting [**12c·F^−^**] and [**12d·F^−^**] complexes exhibited red-shifted circular dichroism (CD), fluorescence, and CPL spectra, underscoring the capability of helicene-based frameworks for anion-responsive chiroptical modulation. These findings highlight how precise structural design and supramolecular engineering can facilitate the development of high-performance, stimuli-responsive chiral luminophores.

**Table 3:** Structures and optical properties of **11a**–**e** and **12a**–**d**.

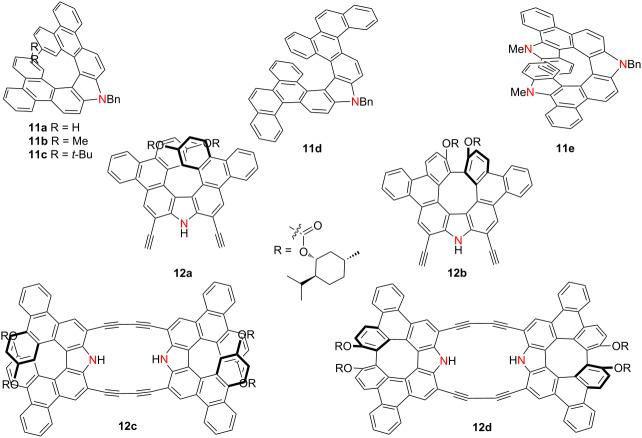						

compound	λ_abs(max)_ [nm]	λ_em_ [nm]	Ф_F_	*|g*_abs_ *|*	|*g*_lum_|	*B*_CPL_ [M^−1^ cm^−1^]

**11a**	418	432, 454	0.28	4.9 × 10^−3^	3.2 × 10^−3^	–
**11b**	419	432, 455	0.27	5.9 × 10^−3^	3.4 × 10^−3^	–
**11c**	419	432, 456	0.31	5.4 × 10^−3^	3.5 × 10^−3^	–
**11d**	422	458, 480	0.10	3.2 × 10^−3^	3.9 × 10^−4^	–
**11e**	412	456	0.24	4.5 × 10^−4^	2.9 × 10^−4^	–
**12a**	436	447, 474	0.45	4.8 × 10^−3^	2.6 × 10^−3^	6.7
**12b**	423	431, 456	0.32	3.8 × 10^−3^	2.2 × 10^−3^	2.8
**12c**	494	502, 536	0.64	2.4 × 10^−3^	6.5 × 10^−4^	19
**12d**	475	485, 514	0.54	2.7 × 10^−3^	1.4 × 10^−3^	31

In 2022, Zhang and co-workers reported a nitrogen-embedded quintuple [7]helicene **13**, constructed by hybridizing helicene and azacorannulene π-systems [26] (Table 4). Compound **13** exhibited distinct absorption bands at 408, 611, and 715 nm, with strong near-infrared (NIR) fluorescence centered at 770 nm and a PLQY value of 0.28. Upon coordination with tris(4-bromophenyl)aminium hexachloroantimonate (BAHA), a new absorption band emerged around 900 nm, extending to 1300 nm, indicative of charge-transfer processes. The enantiomers of **13** displayed mirror-image CD signals and showed excellent dispersibility in polar solvents, highlighting their potential for NIR bio-imaging applications. In parallel, Církva’s group synthesized a series of aza[*n*]helicenes **14a**–**d** via photocyclodehydrochlorination [27]. These compounds exhibited dual fluorescence bands, with emission red-shifting progressively with increasing helical length. Protonation further induced red-shifted emission, with compound **14d**-H^+^ emitting at 542 nm. However, PLQYs decreased significantly from 0.078 to 0.006 with longer helicenes. The CD spectra of **14c** and **14d** were found to resemble their carbohelicene analogues, underscoring the structural fidelity and chiroptical retention upon nitrogen incorporation. Qian’s group developed a series of azahelicenes **15a**–**d** through Bischler–Napieralski cyclization [28]. Notably, compound **15b** displayed a high interconversion barrier of 36.0 kcal mol^−1^, enabling enantiomeric resolution. All compounds exhibited visible-range fluorescence (400–500 nm) and structured UV–vis absorption spectra. Importantly, **15b** showed acid/base-switchable UV and CD spectra, suggesting potential for use in responsive optoelectronic systems. Hu’s group reported an X-shaped double [7]helicene **16** functionalized with four triazole units, which demonstrated absorption at 368 and 516 nm, strong emission at 553 nm, a high PLQY of 0.96, |*g*_abs_| of 1.1 × 10^−2^, |*g*_lum_| of 9.1 × 10^−4^, and *B*_CPL_ of 30.1 M^−1^ cm^−1^ – surpassing the performance of its all-carbon and thiadiazole counterparts [29]. In a related study, Hu’s team synthesized double aza[5]helicenes **17a** and **17b**, among which compound **17b** exhibited red-shifted emission (538–632 nm in CHCl_3_) and the largest Stokes shift (192 nm), attributed to extended conjugation and sulfur incorporation [30] (Table 4). These findings collectively underscore how structural modulation and heteroatom doping can tailor the optical, chiroptical, and stimuli-responsive behavior of azahelicenes, providing strategic design avenues for next-generation chiral optoelectronic materials.

**Table 4:** Structures and optical properties of **13**, **14a**–**d**, **15a**–**d**, **16**, and **17a**,**b**.

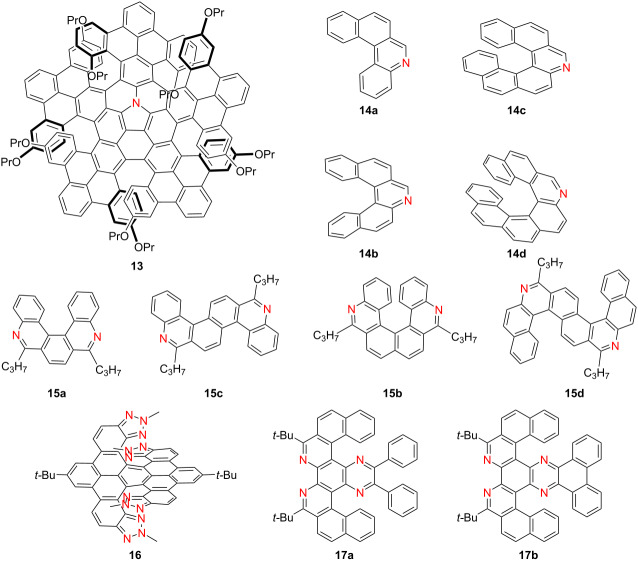						

compound	λ_abs(max)_ [nm]	λ_em_ [nm]	Ф_F_	*|g*_abs_ *|*	|*g*_lum_|	*B*_CPL_ [M^−1^ cm^−1^]

**13**	715	770	0.28	–	–	–
**14a**	313	380, 399	0.077	–	–	–
**14b**	302	410, 431	0.120	–	–	–
**14c**	311	421, 443	0.067	–	–	–
**14d**	337	443, 467	0.029	–	–	–
**15a**	398	408, 430	–	–	–	–
**15b**	404	408, 434	–	–	–	–
**15c**	407	413, 437	–	–	–	–
**15d**	424	434, 456	–	–	–	–
**16**	516	553	0.96	1.1 × 10^−2^	9.1 × 10^−4^	30.1
**17a**	328	458	0.010	–	–	–
**17b**	440	632	0.014	–	–	–

In 2023, Langer’s group synthesized a series of double aza[4,6]helicenes **18a**–**l** featuring diverse peripheral substituents through a one-pot, multistep synthetic protocol [31] (Table 5). Selected compounds such as **18b**, **18c**, **18d** and **18l** exhibit similar λ_abs_ around 410 nm and emit fluorescence centered near 530 nm, demonstrating consistent optical profiles despite structural variation. In a parallel effort, Yang’s group developed an efficient, enantioselective synthetic approach toward azahelicenes via a chiral phosphoric acid-catalyzed multicomponent Povarov reaction or oxidative aromatization [32]. Among the synthesized compounds, compound **19** displayed dual absorption bands at 260 and 325 nm and emission peaks at 420 and 440 nm, which red-shifted to approximately 500 nm upon trifluoroacetic acid treatment. Both the neutral and protonated forms of **19** exhibited mirror-image CD and CPL spectra, with high |*g*_lum_| values of 1.4 × 10^−3^ and 1.3 × 10^−3^, respectively, underscoring their potential for responsive chiral optoelectronic applications. Concurrently, Liu [33] and Ishigaki’s [34] groups independently reported a class of highly twisted nitrogen-doped heptalene derivatives (e.g., compound **20a**), which exhibit consistent absorption at 315 nm and blue fluorescence centered near 450 nm, regardless of the substituents. These compounds display redox and electronic behaviors reminiscent of nitrogen-doped azulenes, featuring strong absorption dissymmetry factors (|*g*_abs_|) at 345 nm – 1.2 × 10^−2^ for compound **20a**, 1.0 × 10^−2^ for **20d**, and 1.3 × 10^−2^ for **20e** (Table 5). Notably, the radical cation form of compound **20e** (**20e**^•+^) exhibits pronounced CD signals extending into the near-infrared region, suggesting potential for redox-responsive chiral photonic systems.

**Table 5:** Structures and optical properties of **18a**–**l**,**19**, and **20a**–**e**.

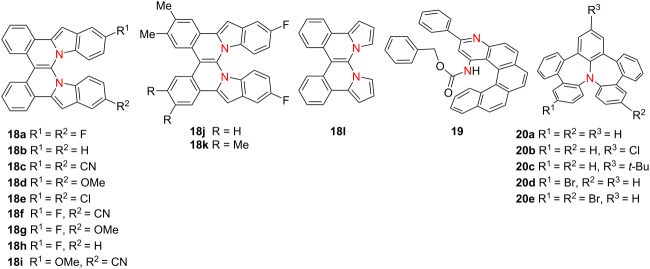					

compound	λ_abs(max)_ [nm]	λ_em_ [nm]	Ф_F_	*|g*_abs_ *|*	|*g*_lum_|

**18b**	411	530	0.15	–	–
**18c**	409	520	0.16	–	–
**18d**	419	525	0.17	–	–
**18i**	413	525	0.14	–	–
**19**	325	420, 440	–	–	1.4 × 10^−3^
**20a**	315^a^, 320^b^	447	–	1.2 × 10^−2^	–
**20b**	315	459	–	–	–
**20c**	315	446	–	–	–
**20d**	320	–	–	1.0 × 10^−2^	–
**20e**	321	–	–	1.3 × 10^−2^	–

^a^Based on reports from Liu's group; ^b^based on reports from Ishigaki's group.

In 2023, Chen’s group reported three nitrogen–nitrogen (NN)-embedded azahelicenes **21a**–**c**, among which compound **21c**, a structurally defined antiaromatic double aza[7]helicene – exhibited distinctive long-wavelength optical and chiroptical properties [35] (Table 6). In the solid state, **21c** emitted in the far-red region at 641 nm (Φ_F_ = 0.10) and demonstrated CPL with |*g*_lum_| = 2.04 × 10^−4^. In solution, **21c** showed a strong absorption band at 560 nm and a high Ф_F_ value of 0.86 at 583 nm, yielding a *B*_CPL_ value of 13.2 M^−1^ cm^−1^. Notably, compound **21c** undergoes reversible redox interconversion to its radical cation **21c**^•+^ and dicationic **21c**^2+^ states via chemical oxidation, enabling controllable switching between antiaromatic and aromatic configurations. These results provide a compelling strategy for engineering redox-switchable chiral luminophores. In 2024, the same research group expanded on this redox-responsive platform by constructing a polycationic open-shell cyclophane **22**, comprising carbazole-embedded aza[7]helicene subunits [36]. Compound **22** displays intense fluorescence (Φ_F_ = 0.99), exceptionally high *B*_CPL_ as 100.2 M^−1^ cm^−1^, and marked chiroptical activity (|*g*_abs_| = 2.50 × 10^−3^ at 435 nm; |*g*_lum_| = 5.00 × 10^−3^ at 460 nm) (Table 6). Upon mild oxidation, neutral **22** undergoes stepwise conversion into highly charged, multispin open-shell species **22**^2+2•^ and **22**^4+4•^, preserving strong chiroptical signals. This study presents a novel approach to constructing stable, redox-switchable chiral luminophores based on extended azahelicene architectures, offering broad potential for molecular electronics and spintronic devices.

**Table 6:** Structures and optical properties of **21a**–**c** and **22**.

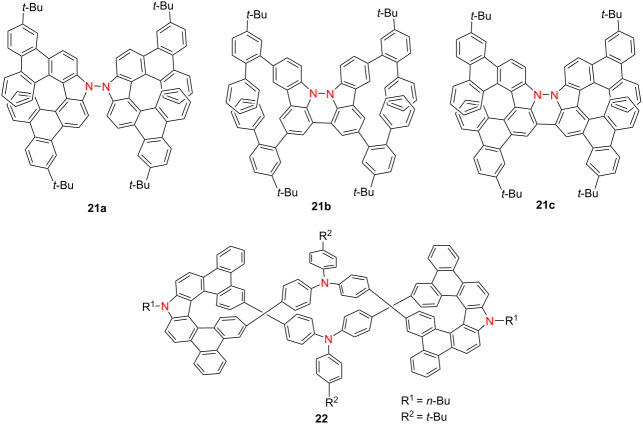						

compound	λ_abs(max)_ [nm]	λ_em_ [nm]	Ф_F_	*|g*_abs_ *|*	|*g*_lum_|	*B*_CPL_ [M^−1^ cm^−1^]

**21a**	408	423	0.26	9.78 × 10^−4^	–	–
**21a** in film	≈410	449	0.15	–	–	–
**21b**	495	521	0.77	–	–	–
**21b** in film	≈500	548	0.63	–	–	–
**21c**	560	583	0.86	4.76 × 10^−4^	2.22 × 10^−4^	13.2
**21c** in film	≈570	641	0.10	–	2.04 × 10^−4^	–
**22**	438	480	0.99	2.50 × 10^−3^	5.00 × 10^−3^	100.2

In 2024, Qiu’s group synthesized π-extended diaza[7]helicenes **23a**–**f** incorporating dual heptagonal rings [37]. Compound **23a** exhibits dynamic chirality, aggregation-induced emission (AIE), and intense CPL (|*g*_lum_| = 1.7 × 10^−2^), whereas compound **23f**, with lateral π-extension, shows enhanced thermal stability and green emission at 517 nm (Table 7). Kuehne and co-workers reported two radical aza[7]helicenes, **24a** and **24b**, exhibiting distinct photophysical behaviors [38]. Compound **24b** features a higher PLQY (0.43), while **24a** demonstrates doublet-state CPL (|*g*_lum_| = 5.0 × 10^−4^), highlighting the potential of helicene radicals for spintronic applications. Meng’s group synthesized carbonyl-nitrogen embedded hetero[7]helicenes **25a** and **25b** bearing axial chirality [39]. Compound **25a** displays excellent optical characteristics with Φ_F_ = 0.57, |*g*_abs_| = 1.7 × 10^−2^, |*g*_lum_| = 1.4 × 10^−3^, and a *B*_CPL_ of 8.94 M^−1^ cm^−1^. Then, Chen’s group contributed triple aza[6]helicenes **26a** and **26b** with |*g*_lum_| values of approximately 3.0 × 10^−3^, offering new architectures for CPL-active helicenes [40]. Singh’s group developed fluorophore-conjugated aza[7]helicenes **27a**–**d**, with **27b** demonstrating pronounced intramolecular charge transfer (ICT), a high Φ_F_ of 0.71 and an extended fluorescence lifetime (*τ*) of 15.5 ns [41]. Wu’s group synthesized a family of expanded azahelicenes **28a**–**e**, where increasing helical length leads to red-shifted emission, prolonged lifetime, and attenuated PLQY [42]. Nonetheless, these compounds exhibit outstanding chiroptical performance, with |*g*_abs_|_max_ reaching 4.8 × 10^−2^, |*g*_lum_|_max_ = 2.1 × 10^−2^, and *B*_CPL_ values up to 76 M^−1^ cm^−1^. Collectively, these investigations underscore the efficacy of heteroatom doping, extended π-conjugation, and radical design in advancing azahelicene-based systems. These approaches significantly enhance optical and chiroptical performance, paving the way for high-efficiency chiral optoelectronic and quantum materials.

**Table 7:** Structures and optical properties of **23a**–**f**, **24a**,**b**, **25a**,**b**, **26a**,**b**, **27a**–**d**, and **28a**–**e**.

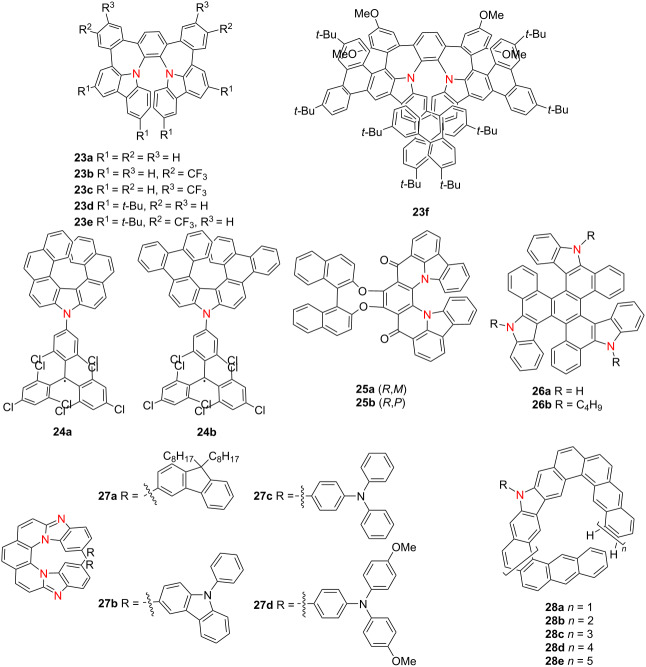						

compound	λ_abs(max)_ [nm]	λ_em_ [nm]	Ф_F_	*|g*_abs_ *|*	|*g*_lum_|	*B*_CPL_ [M^−1^ cm^−1^]

**23a**	360	625	–	–	1.7 × 10^−2a^	–
**23f**	462	517	–	–	2.0 × 10^−3^	–
**24a**	642	696	0.34	4.4 × 10^−4^	5 × 10^−4^	0.25
**24b**	655	712	0.43	1 × 10^−4^	–	–
**25a**	506	525	0.57	1.7 × 10^−2^	1.4 × 10^−3^	8.94
**25b**	513	535	0.55	2.2 × 10^−2^	8 × 10^−4^	4.29
**26a**	388	506, 530	0.055	1.2 × 10^−2^	3.0 × 10^−3^	–
**26b**	393	508, 532	0.058	1.4 × 10^−2^	3.2 × 10^−3^	–
**27a**	483	524	0.38	–	–	–
**27b**	487	539	0.71	–	–	–
**27c**	459	590	0.24	–	–	–
**27d**	470	611	0.53	–	–	–
**28a**	414	496, 532	0.152	–	–	–
**28b**	≈475	511, 543	0.116	4.4 × 10^−2^	3 × 10^−3^	16
**28c**	≈475	522, 550	0.089	4.8 × 10^−2^	1.4 × 10^−2^	61
**28d**	≈475	530, 554	0.066	4.3 × 10^−2^	2.1 × 10^−2^	76
**28e**	≈475	530, 555	0.034	–	–	–

^a^In the aggregated state.

In 2024, Kivala’s group selectively synthesized highly distorted [6]helicenes **29a** and **29b** incorporating azocine units via a regioselective Beckmann rearrangement from oxime precursor **29c** [43] (Table 8). For comparative evaluation, the corresponding lactams **29d** and **29e** and amines **29f** and **29g** were also obtained. Compounds **29a** and **29b** exhibit λ_abs_ centered at 513 nm, while the amines **29f** and **29g** display high Ф_F_ values of 0.48 and 0.56, respectively. Notably, azocine derivative **29b** exhibits the highest CPL activity among the series, with a |*g*_lum_| value of 1.6 × 10^−3^. In addition, both **29a** and **29b** demonstrate redox activity, undergoing reversible formation of radical anions, dianions, and radical cations. The radical cation **29b**^•+^, in particular, exhibits a broad near-infrared (NIR) absorption band extending to 3000 nm, highlighting its potential for NIR optoelectronic applications. Building on this work, in 2025 the same group reported the synthesis of a stable N-heterotriangulene dimer (compound **30**) bridged by a rigid π-conjugated [5]helicene [44]. This chiral dimer undergoes reversible stepwise oxidation to **30**^•+^ and **30**^2+^, accompanied by pronounced NIR Cotton effects extending up to 2000 nm. These results provide critical insights into the rational design of redox-switchable, NIR-active chiral molecular systems, underscoring their promise in advanced optoelectronic and spintronic technologies.

**Table 8:** Structures and optical properties of **29a**–**f** and **30**.^a^

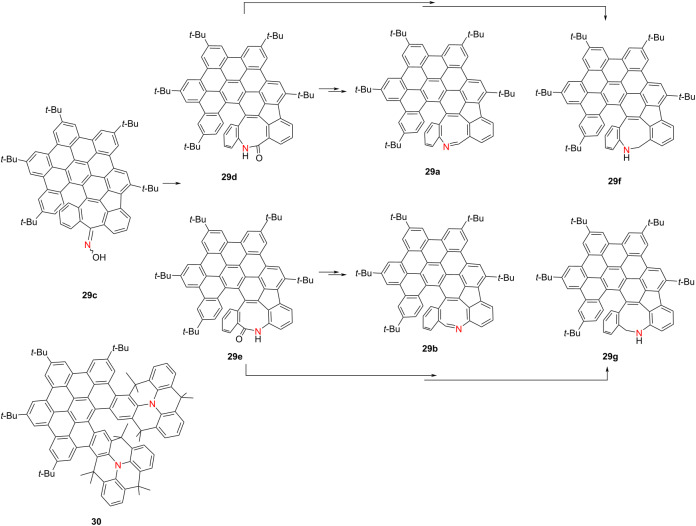						

compound	λ_abs(max)_ [nm]	λ_em_ [nm]	Ф_F_	*|g*_abs_ *|*	|*g*_lum_|	*B*_CPL_ [M^−1^ cm^−1^]

**29a**	513	540, 565	0.01	2.5 × 10^−3^	–	–
**29b**	513	552, 582	0.12	1.9 × 10^−3^	1.6 × 10^−3^	–
**29d**	510	539, 570	0.52	3.0 × 10^−3^	6.0 × 10^−4^	–
**29e**	510	543, 575	0.51	2.1 × 10^−3^	2.4 × 10^−4^	–
**29f**	510	536, 570	0.48	2.0 × 10^−3^	9.1 × 10^−4^	–
**29g**	547	609, 652	0.56	2.4 × 10^−3^	6.0 × 10^−4^	–
**30**	495	534	0.42	1.25 × 10^−3^	1.1 × 10^−3^	7.00

^a^The optical properties of compound **29c** are not mentioned in the original paper.

In 2024, Tanaka’s group synthesized and characterized a series of length-variable aza[*n*]helicenes **31a**–**f** via a one-pot intramolecular cyclodehydrogenation [45] (Table 9). Notably, compounds **31e** and **31f** represent the first examples of triple-layered heterohelicenes with fully conjugated frameworks. All members of the series demonstrate high solubility, attributed to intermolecular hydrogen bonding with solvent molecules. With increasing helical length, both the λ_abs_ and λ_em_ exhibit progressive bathochromic shifts, while the Ф_F_ values systematically decline, without clear saturation within the investigated range. Chiroptical measurements of the *N*-butylated aza[*n*]helicenes **31g**–**j** reveal |*g*_abs_| and |*g*_lum_| values on the order of 10^−3^. These findings address long-standing challenges in the synthesis and stabilization of extended heterohelicenes, paving the way for the development of structurally persistent, π-extended chiral materials. In a parallel effort, Tanaka’s group synthesized benzannulated double aza[9]helicene **32a** and its alkylated derivatives **32b** and **32c** via a one-pot oxidative fusion strategy [46]. Compared to the parent compound **32a** (Φ_F_ = 0.07), compounds **32b** and **32c** exhibit significantly enhanced Φ_F_ (0.35), red-shifted absorption bands, and |*g*_abs_| values of 2.4 × 10^−3^ and 2.3 × 10^−3^ at 345 nm, respectively. Their corresponding *B*_CPL_ values reach 16.0 and 19.2 M^−1^ cm^−1^. Furthermore, terminus-functionalized aza[9]helicenes **33a**, **33b**, and **33c** were prepared to investigate interlayer interactions [47]. Among them, the pyrene-decorated compound **33c** displays red-shifted emission and prolonged fluorescence lifetimes as solvent polarity increases, indicating enhanced excited-state stabilization. Collectively, these studies offer valuable strategies for stabilizing long π-extended helicenes and finely tuning their chiroptical and emissive properties, thereby advancing their application in multifunctional chiral photonic and sensing platforms.

**Table 9:** Structures and optical properties of **31a**–**j**, **32a**–**c**, and **33a**–**c**.

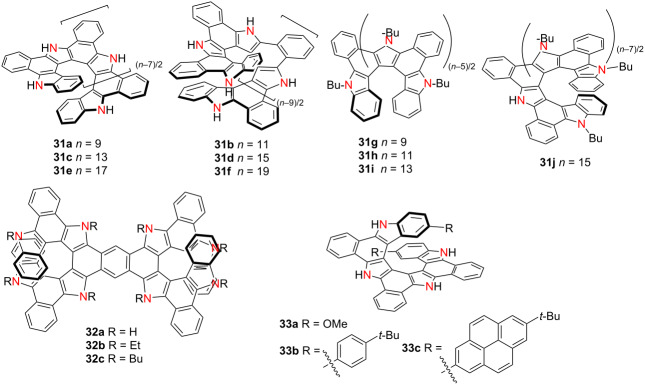						

compound	λ_abs(max)_ [nm]	λ_em_ [nm]	Ф_F_	*|g*_abs_ *|*	|*g*_lum_|	*B*_CPL_ [M^−1^ cm^−1^]

**31a**	412	437, 466, 500	0.21	–	–	–
**31b**	425	452, 479, 514	0.17	–	–	–
**31c**	438	427, 450, 480	0.11	–	–	–
**31d**	451	466, 491, 530	0.09	–	–	–
**31e**	388	483, 511	0.18	–	–	–
**31f**	310	508	0.08	–	–	–
**31g**	409	465, 495	0.16	5.6 × 10^−3^	4.5 × 10^−3^	8.6^a^
**31h**	314	482, 508	0.16	4.2 × 10^−3^	4.2 × 10^−3^	–
**31i**	315	508	0.09	4.2 × 10^−3^	1.7 × 10^−3^	–
**31j**	≈385	≈520	0.07	1.7 × 10^−3^	5.7 × 10^−3^	–
**32a**	464	496, 529, 570	0.07^b^ 0.33^c^	–	–	–
**32b**	510	521, 555	0.35	2.4 × 10^−3^	–	16.0
**32c**	508	522, 556	0.35	2.3 × 10^−3^	–	19.2
**33a**	415	441, 466, 500	0.19	–	–	–
**33b**	414	437, 466, 500	0.21	–	–	–
**33c**	416	441, 466, 500	0.08	–	–	–

^a^According to reference paper [42]; ^b^in THF; ^c^in DMSO.

In 2025, Gryko’s group synthesized a series of heterohelicenes **34a**–**c**, featuring a 1,4-dihydropyrrolo[3,2-*b*]pyrrole (DHPP) core [48] (Table 10). The compounds exhibit similar absorption and emission profiles. However, compound **34c** stands out due to its pronounced solvatofluorochromism (λ_em_ = 546 nm, Φ_F_ = 0.42 in DMSO). Among the series, compound **34b** exhibits the highest |*g*_lum_| of 7.22 × 10^−3^, while compound **34c** shows the greatest *B*_CPL_ as 29.3 M^−1^ cm^−1^. These studies underscore the importance of regioisomerism and molecular core design in optimizing the chiroptical and emissive properties of heteroatom-rich nanographenes, advancing their potential in next-generation optoelectronic and chiral photonic devices.

**Table 10:** Structures and optical properties of **33a**,**b** and **34a**–**c**.

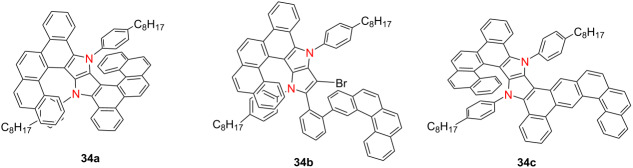						

compound	λ_abs(max)_ [nm]	λ_em_ [nm]	Ф_F_	*|g*_abs_ *|*	|*g*_lum_|	*B*_CPL_ [M^−1^ cm^−1^]

**34a**	438	460, 481	0.270	–	1.33 × 10^−3^	2.0
**34b**	446	463, 488	0.045	–	6.11 × 10^−3^	4.3
**34c**	456	483, 505	0.324	–	3.25 × 10^−3^	29.3

### B,N-containing helicenes

Enhancing charge transfer between electron-donating and electron-accepting units, as well as extending π-conjugated frameworks, are widely employed strategies for achieving longer-wavelength emission in optoelectronic materials. Inspired by the electronic configuration of borazine, boron has emerged as a valuable electron-accepting counterpart to electron-donating nitrogen in conjugated systems, enabling the design of donor–acceptor helicenes with tunable photophysical properties.

In 2020, Ema and co-workers developed a series of chiral carbazole-based BODIPY analogues **35a**–**f**, derived from helical carbazole-based BF_2_ dyes [49] (Table 11). These analogues exhibit red-shifted emission and enhanced CPL compared to their carbazole-based helicene precursors. At λ_abs_ (≈500 nm), the compounds display |*g*_abs_| values ranging from 1.1 × 10^−3^ to 3.1 × 10^−3^, Ф_F_ values of 20–36%, and |*g*_lum_| values between 7.0 × 10^−4^ and 1.9 × 10^−3^. In a subsequent study, Ema’s group reported an *N*-containing hetero[7]helicene **36a** containing a boron–nitrogen coordination site [50]. Its chiroptical properties could be modulated through the addition of tetrabutylammonium (TBA) salts, which transformed the boron center from a trigonal planar to a tetrahedral geometry, thereby enhancing the |*g*_lum_| from 4.7 × 10^−4^ to 1.5 × 10^−3^ (OAc^−^, **36c**) and 1.7 × 10^−3^ (F^−^/OH^−^, **36b**/**36d**). Treatment with Ag^+^ ions reversed this coordination, restoring the neutral trigonal boron center and its initial optical characteristics. These findings underscore the potential of boron–nitrogen-embedded helicene frameworks as tunable chiral luminophores with reversible CPL modulation, offering promising strategies for the development of advanced molecular optoelectronic devices.

**Table 11:** Structures and optical properties of **35a**–**f** and **36a–d**.

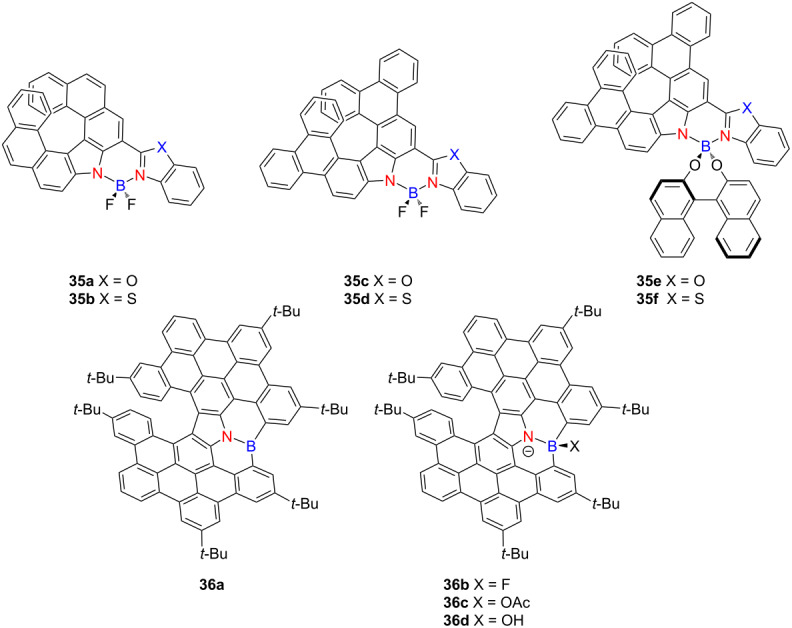						

compound		λ_abs(max)_ [nm]	λ_em(max)_ [nm]	Ф_F_	*|g*_abs_ *|*	|*g*_lum_|

**35a**		495	568	0.22	2.7 × 10^−3^	1.7 × 10^−3^
**35b**		508	594	0.20	3.1 × 10^−3^	1.3 × 10^−3^
**35c**		508	566	0.33	1.2 × 10^−3^	8.7 × 10^−4^
**35d**		524	592	0.21	1.1 × 10^−3^	7.0 × 10^−4^
**35e**	(*R*,*P*)	508	576	0.30	2.3 × 10^−3^	1.5 × 10^−3^
	(*R*,*M*)	509	571	0.36	1.5 × 10^−3^	1.2 × 10^−3^
**35f**	(*R*,*P*)	530	605	0.20	1.8 × 10^−3^	1.2 × 10^−3^
	(*R*,*M*)	532	602	0.26	1.5 × 10^−3^	8.8 × 10^−4^
**36a**		487	493	–	1.6 × 10^−3^	4.7 × 10^−4^
**36b**		502	512	–	3.0 × 10^−3^	1.7 × 10^−3^
**36c**		510	526	–	2.9 × 10^−3^	1.5 × 10^−3^
**36d**		511	520	–	3.2 × 10^−3^	1.7 × 10^−3^

In 2021, Hatakeyama and co-workers developed an expanded B,N-containing heterohelicene **37** via a one-step synthesis employing excess BBr_3_ at 180 °C in an autoclave, achieving a 44% yield [51] (Table 12). In a 1 wt % PMMA-dispersed film, compound **37** exhibited ultra-narrowband emission (FWHM = 16 nm) at 484 nm with an 80% PLQY. OLEDs based on **37** demonstrated excellent external quantum efficiency, current efficiency, and power efficiency. Duan and co-workers reported B,N-containing double hetero[7]helicenes **38a**,**b**, which exhibited deep-red fluorescence emission at 662 and 692 nm, respectively, with narrow emission bandwidths (full width at half maximum, FWHM = 38 nm) and exceptional PLQYs of 100% [52]. Remarkably, they achieved maximum EQEs of 28.1% and 27.6%, representing the highest reported values for thermally activated delayed fluorescence (TADF) emitters operating above 650 nm. Shortly thereafter, Wang’s group reported a related series of B,N-containing compounds **38a**–**c**, which displayed pronounced chiroptical activity in the visible region [53]. These compounds displayed the highest |*g*_abs_| values recorded for helicenes to date – 0.033, 0.031, and 0.026 at 502, 518, and 526 nm, respectively. They also showed near-unity Ф_F_ values of 100%, 99%, and 90%, with corresponding λ_em_ at 660, 684, and 696 nm, and |*g*_lum_| values of 2 × 10^−3^. The calculated *B*_CPL_ reached 28.5, 37.1, and 40.0 M^−1^ cm^−1^, positioning these helicenes among the most efficient red CPL emitters reported to date (Table 12).

**Table 12:** Structures and optical properties of **37**, **38a–c**, and **39**.

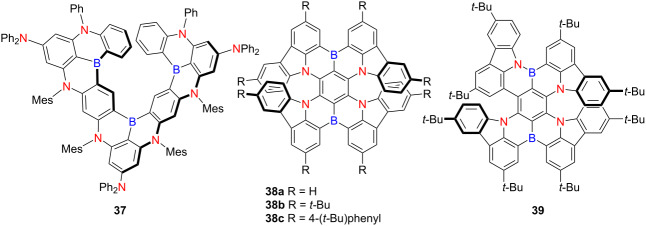					

compound	λ_abs(max)_ [nm]	λ_em(max)_ [nm]	Ф_F_	*|g*_abs_ *|*	|*g*_lum_|

**38a**	627	660	1.00	3.3 × 10^−2^	2.0 × 10^−3^
**38b**	650	684	0.99	3.1 × 10^−2^	2.0 × 10^−3^
**38c**	662	696	0.90	2.6 × 10^−2^	2.0 × 10^−3^
**39**	590	617	0.96	1.2 × 10^−2^	1.4 × 10^−3^

film		λ_abs(max)_ [nm]	λ_em(max)_ [nm]	Ф_F_	FWHM [nm]

**37** in PMMA		477	484	0.80	16
**38a** in CBP		–	672	–	48
**38b** in CBP		–	698	–	49
**39** in mCPBC		–	624	0.95	–

device	λ_EL(max)_ [nm]	|*g*_EL_|	FWHM [nm]	CIE coordinate	EQE_max_ [%]

**37**	480	–	17	(0.09, 0.21)	22.9^a^
**38a**	664	–	48	(0.72, 0.28)	28.1
**38b**	686	–	49	(0.72, 0.28)	27.6
**39**	617	1.9 × 10^−3^	48	(0.67, 0.33)	36.6

^a^As detected at 10 cd m^−2^.

However, such long-wavelength emission poses challenges for achieving optimal color purity in OLED devices. To overcome this limitation, Duan’s group subsequently introduced a covalent B–N bond into the helicene framework in 2023, affording compound **39** [54]. This material emits at 617 nm with a FWHM of 38 nm and maintains a near-unity PLQY. Circularly polarized OLEDs (CP-OLEDs) based on **39** exhibit outstanding device performance, achieving a |*g*_EL_| of 1.91 × 10^−3^, a record-high EQE exceeding 36%, and operational stability with an LT_95_ of approximately 400 h at 10,000 cd m^−2^. These findings underscore the efficacy of B–N covalent integration in helicene-based frameworks for realizing high-efficiency, spectrally optimized, and robust red CP-OLED emitters.

In 2022, Yang and co-workers reported a W-shaped double hetero[5]helicene **40**, incorporating boron, nitrogen, and sulfur atoms within its framework [55] (Table 13). Compound **40** exhibits exceptional photophysical and electroluminescent performance, including a PLQY value of 100% and a |*g*_lum_| value of 2.1 × 10^−3^. Circularly polarized organic light-emitting diodes (CP-OLEDs) based on **40** demonstrated a |*g*_EL_| of 2.2 × 10^−3^, a narrow emission bandwidth (FWHM = 49 nm), and a maximum external quantum efficiency (EQE) of 31.5%, placing it among the highest-performing multiple-resonance-induced thermally activated delayed fluorescence (MR-TADF) emitters to date. In 2023, the same group introduced the first deep-blue chiral MR-TADF emitters based on heterohelicene scaffolds **41a**–**c** [56]. These compounds exhibited sharp emissions at 440–444 nm in solution and 445–449 nm in doped films, with emission bandwidths as narrow as 23 nm and PLQYs reaching up to 95%. Notably, racemic **41b** and **41c** displayed excellent chiroptical properties, with |*g*_lum_| values ranging from 1.4 to 1.5 × 10^−3^ and *B*_CPL_ values exceeding 22 M^−1^ cm^−1^. Compound **41c**, in particular, achieved a |*g*_EL_| of 2.6 × 10^−3^ and a maximum luminance exceeding 10,000 cd m^−2^. These findings underscore the significant potential of heteroatom-integrated helicene systems as high-efficiency, CPL-active MR-TADF materials for next-generation OLED technologies, particularly in the development of deep-blue emissive devices with high color purity and device efficiency.

**Table 13:** Structure and optical properties of **40** and **41a**–**c**.

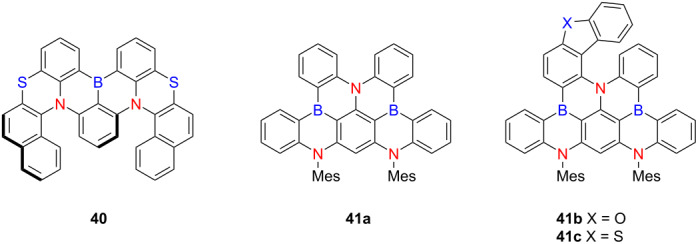						

compound	λ_abs(max)_ [nm]	λ_em(max)_ [nm]		Ф_F_	*|g*_abs_ *|*	|*g*_lum_|

**40**	483	520		0.98	–	2.1 × 10^−3^
**41a**	424	440		0.82	–	–
**41b**	422	443		0.91	1.4 × 10^−3^	1.4 × 10^−3^
**41c**	427	444		0.95	1.5 × 10^−3^	1.5 × 10^−3^

film	λ_abs(max)_ [nm]	λ_em(max)_ [nm]		Ф_F_	FWHM [nm]	

**40** in DMIC-TRZ	–	525		–	48	
**41a** in DOBNA-OAr	–	445		0.82	35	
**41b** in DOBNA-OAr	–	448		0.91	28	
**41c** in DOBNA-OAr	–	449		0.95	28	

device	λ_EL(max)_ [nm]	|*g*_EL_|	FWHM [nm]	CIE coordinate		EQE_max_ [%]

**40**	524	2.2 × 10^−3^	49	(0.26, 0.66)		31.5
**41a**	443	–	26	(0.15, 0.05)		23.4
**41b**	445	2.2 × 10^−4^	24	(0.15, 0.04)		27.5
**41c**	447	2.6 × 10^−4^	24	(0.15, 0.05)		29.3

In 2022, Marder and co-workers introduced various boryl substituents at both termini of a series of nitrogen-doped [5]helicenes, yielding helicenoids **42a**–**h** [57] (Table 14). The Bpin-substituted derivatives **42a**–**e** exhibited broad emission across the 400–800 nm range, whereas their analogues **42f** and **42g** showed negligible emission, indicating a strong dependence of photophysical behavior on boryl-substituent identity. Compared to their parent azahelicenes, these compounds displayed significantly larger Stokes shifts, highlighting the pronounced electronic effects of boryl incorporation. Notably, when a CF_3_ group was introduced as a substituent on the azahelicene core, the resulting boryl-functionalized compound **42c** exhibited an emission maximum at 563 nm in CH_2_Cl_2_, with a quantum yield of 15%, representing the highest emission efficiency observed among the boron-containing quasi-circulenes.

**Table 14:** Structure and optical properties of **42a**–**h**.^a^

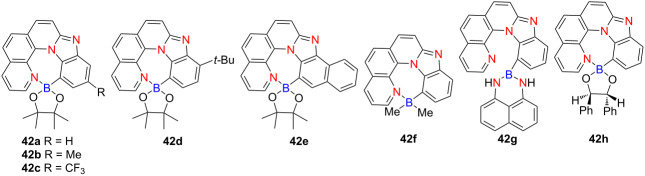			

compound	λ_abs(max)_ [nm]	λ_em(max)_ [nm]	Ф_F_

**42a**	372	520	0.08
**42b**	373	522	0.08
**42c**	364	563	0.15
**42d**	372	530	0.07
**42e**	407	588	0.05
**42f**	385	–	–
**42g**	366	–	–
**42h**	–	–	–

^a^No *g*_abs_ or *g*_lum_ values were reported.

In 2022, Lu and co-workers developed a series of helical aza-BODIPY analogues **43a**–**h**, featuring a distinctive B–O–B bridge installed within each molecule [58] (Table 15). These compounds display broad chiroptical responses extending from the ultraviolet to the entire visible spectrum – an uncommon characteristic among helicene-type systems. Among them, the phenyl-substituted aza[7]helicene **43f** exhibits pronounced chiroptical activity, with |*g*_abs_| and |*g*_lum_| values reaching 3.04 × 10^−3^ and 1.30 × 10^−3^, respectively, and a high *B*_CPL_ of 11.5 M^−1^ cm^−1^ in the near-infrared region. In contrast, the corresponding aza[5]helicene analogue shows negligible chiral response, with |*g*_abs_| and |*g*_lum_| values in the 10^−5^ range. To further enhance chiroptical performance, Lu’s group introduced edge-positioned methyl and ethyl substituents into the helical core, affording **44a** and **44b** [59]. Compared with **43c**, they are with significantly improved |*g*_abs_| values of 1.51 × 10^−3^ and 1.69 × 10^−3^, respectively. This study underscores the critical importance of molecular design in modulating chiroptical properties and provides valuable insights into the development of helicene-based BODIPY systems for near-infrared CPL applications. In 2024, Shimizu’s group reported azabora[6]helicenes **45a** and **45b** [60]. However, their enantiomers could not be isolated due to low racemization barriers. The F- and Ph-coordinated derivatives displayed moderate PLQYs in solution (0.26 and 0.18, respectively), which dropped markedly in the solid state (0.02 and 0.04) owing to aggregation-caused quenching (ACQ).

**Table 15:** Structure and optical properties of **43a**–**h**, **44a**,**b**, and **45a**,**b**.

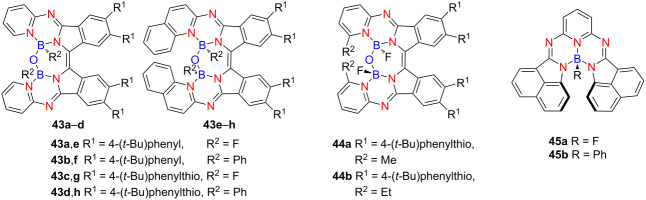					

compound	λ_abs(max)_ [nm]	λ_em(max)_ [nm]	Ф_F_	*|g*_abs_ *|*	|*g*_lum_|

**43a**	588	625	0.59	4 × 10^−5^	3 × 10^−5^
**43b**	623	649	0.56	–	–
**43c**	601	640	0.31	–	–
**43d**	634	668	0.12	–	–
**43e**	646	682	0.30	2.0 × 10^−3^	1.3 × 10^−3^
**43f**	677	708	0.24	3.0 × 10^−3^	1.3 × 10^−3^
**43g**	660	695	0.16	1.8 × 10^−3^	1.2 × 10^−3^
**43h**	691	719	0.10	–	–
**44a**	624	665	0.08	1.5 × 10^−3^	–
**44b**	625	665	0.07	1.7 × 10^−3^	–
**45a**	548	568	0.26	–	–
**45b**	554	574	0.18	–	–

In 2023, Yang and co-workers reported a pair of (NBN)_2_-containing double and quadruple helicenes **46a**–**d** [61] (Table 16). The neutral compounds exhibited high PLQYs of 99% and 65% in solution, and 90% and 55% in PMMA-doped films, respectively, with exceptionally narrow full-width (FWHM values as 24 nm and 22 nm). Stepwise titration experiments with fluoride ions induced a change in the coordination number of the boron centers from three to four, forming corresponding anionic species. This coordination triggered red-shifted absorption and CPL responses while maintaining excellent PLQYs – 99% and 90% in solution, and 80% and 77% in PMMA-doped films, respectively.

**Table 16:** Structure and optical properties of **46a**–**d**.

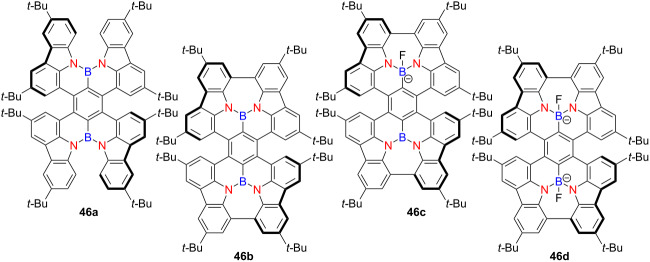					

compound	λ_abs(max)_ [nm]	λ_em(max)_ [nm]	Ф_F_	*|g*_abs_ *|*	|*g*_lum_|

**46a** ^a^	511	524	0.99	–	–
**46b** ^a^	507	522	0.65	6.2 × 10^−3^	1.0 × 10^−3^
**46c** ^b^	524	567	0.99	5.0 × 10^−3^	6.0 × 10^−4^
**46d** ^b^	518	541	0.90	6.0 × 10^−3^	7.0 × 10^−4^

film		λ_abs(max)_ [nm]	λ_em(max)_ [nm]	Ф_F_	FWHM [nm]

**46a** in PMMA		–	–	0.95	–
**46b** in PMMA		–	–	0.55	–
**46c** in PMMA		–	–	0.80	–
**46d** in PMMA		–	–	0.77	–

^a^In toluene; ^b^in acetone.

In 2024, Wang’s group developed a B,N-embedded hetero[8]helicene **47**, exhibiting narrow green emission at 531 nm (FWHM = 36 nm), a high PLQY of 93%, and outstanding CP-OLED performance (EQE = 32.0%; |*g*_EL_| = 7.74 × 10^−4^) [62] (Table 17). Bin’s group introduced orthogonal spiro-structures into hetero[6]helicenes **48a**–**c**, achieving near-unity PLQYs in solution (up to 99%) and OLED external quantum efficiencies (EQEs) exceeding 31% [63]. Chen’s group reported **49**, a B,N-containing hetero[9]helicene that emits at 578 nm with a PLQY of 98% and showing excellent chiroptical properties (|*g*_lum_| = 5.8 × 10^−3^; *B*_CPL_ = 220.75 M^−1^ cm^−1^) [64]. OLEDs incorporating compound **49** demonstrated an EQE of 35.5% and |*g*_EL_| = 6.2 × 10^−3^. Zhang’s group synthesized **50a**–**f**, with and without installed heptagons [65]. The heptagon-containing derivatives showed red-shifted emission, broader FWHM, lower PLQYs, and diminished *B*_CPL_ values, indicating a trade-off between extended conjugation and emissive efficiency. Yin’s group introduced 1,4-BN motifs into compounds **51a** and **51b**, which emitted blue-green light at 474 and 465 nm, respectively, and exhibited moderate CPL activity (|*g*_lum_| ≈ 5 × 10^−4^) [66] . OLEDs based on compound **51a** emitted at 502 nm and achieved an EQE of 3.18%. Liu’s group positioned B and N atoms on the inner rim of **52a** and **52b** [67]. While **52b** exhibited remarkably high |*g*_abs_| and |*g*_lum_| values (6.1 × 10^−2^ and 2.4 × 10^−2^, respectively), its PLQY was relatively low (24%). Further molecular optimization led to the development of compounds **53a**–**c**, which demonstrated ultra-narrow emission bands (FWHM = 16–34 nm), high PLQYs (67–82%), and exceptional CPL brightness (*B*_CPL_s of 583, 374, and 349 M^−1^ cm^−1^, respectively), with compound **53a** setting a new record for BN-containing helicene CPL brightness [68]. These collective findings underscore the critical role of rational BN doping, π-conjugation engineering, and structural rigidity in precisely tuning the photophysical and chiroptical properties of helicene-based materials, thereby advancing the design of next-generation CPL-active optoelectronic systems with superior performance metrics.

**Table 17:** Structure and optical properties of **47**, **48a**–**c**, **49**, **50a**–**f**, **51a**,**b**, **52a**,**b**, and **53a**–**c**.

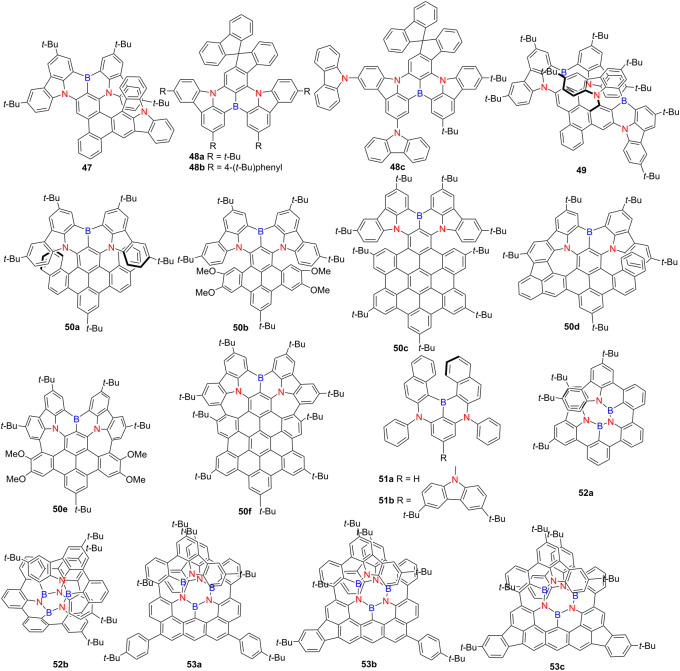					

compound	λ_abs(max)_ [nm]	λ_em(max)_ [nm]	Ф_F_	*|g*_abs_ *|*	|*g*_lum_|

**47**	510	531	0.93	1.4 × 10^−3^	5.8 × 10^−4^
**48a**	482	503	0.91	–	–
**48b**	495	516	0.99	–	–
**48c**	493	515	0.94	–	–
**49**	546	578	0.98	5.6 × 10^−3^	5.8 × 10^−3^
**50a**	548	595	0.68	7.4 × 10^−3^	2.7 × 10^−3^
**50b**	545	585	0.66	8.6 × 10^−3^	2.5 × 10^−3^
**50c**	553	598	0.74	3.1 × 10^−3^	2.7 × 10^−3^
**50d**	622	675	0.11	4.7 × 10^−3^	2.9 × 10^−3^
**50e**	563	623	0.27	–	–
**50f**	595	641	0.02	6.6 × 10^−3^	5.0 × 10^−3^
**51a**	453	474	0.83	6.2 × 10^−3^	5.1 × 10^−4^
**51b**	447	465	0.54	2.5 × 10^−3^	4.8 × 10^−4^
**52a**	403	409	0.31	3.6 × 10^−2^	2.4 × 10^−2^
**52b**	423	430	0.24	6.1 × 10^−2^	4.8 × 10^−2^
**53a**	506	515	0.82	2.4 × 10^−2^	1.7 × 10^−2^
**53b**	513	529	0.67	1.1 × 10^−2^	1.2 × 10^−2^
**53c**	516	535	0.72	1.1 × 10^−2^	8.0 × 10^−3^

film	λ_abs(max)_ [nm]	λ_em(max)_ [nm]	Ф_F_		FWHM [nm]

**46a** from CHCl_3_	–	667	0.02		48
**46b** from CHCl_3_	–	632	0.04		35
**47** in PhCzBCz	–	≈545	0.92		≈50
**51a** in DPEPO	–	472	0.32		38
**51b** in DPEPO	–	467	0.42		29

device	λ_EL(max)_ [nm]	|*g*_EL_|	FWHM [nm]	CIE coordinate	EQE_max_ [%]

**47**	536	7.7 × 10^−4^	38	(0.32, 0.66)	31.1
**48a**	490	–	30	(0.10, 0.41)	25.2
**48b**	506	–	37	(0.15, 0.65)	29.2
**48c**	522	–	37	(0.22, 0.70)	31.0
**49**	580	6.2 × 10^−3^	48	(0.53, 0.46)	35.4
**51a**	502	–	35	(0.14, 0.55)	3.2

However, these findings also suggest that boron may not always be the optimal choice for enhancing charge-transfer properties. The delocalization of electrons between the vacant p-orbital of boron and the electron-rich π-conjugated systems can diminish both the electron-accepting capability of boron and the electron-donating efficiency of the conjugated framework. Additionally, the inherently low electronegativity of boron further limits its effectiveness as an electron acceptor, thereby restricting the achievable red-shift in emission. To overcome these limitations, alternative electron-withdrawing atoms and functional groups have been introduced into nitrogen-doped helicene frameworks to improve their photophysical performance and extend emission into the longer wavelength region.

### X,N-containing helicenes (X = O, S or Se)

Imide functional groups are well recognized for their strong electron-accepting character, making them valuable moieties in the design of optoelectronic materials. When incorporated into π-conjugated frameworks, imide groups can significantly modulate electronic structures and enhance properties such as fluorescence efficiency, charge transport, and chiroptical responses. In this section, we begin by summarizing representative imide-functionalized helicenes, highlighting their structural features and photophysical performances. In 2020, Ravat’s group introduced a novel class of helically chiral diimide molecules **54a**–**c**, which integrate the favorable characteristics of arylene diimides within the chiral architecture of [*n*]helicenes [69]. These compounds exhibit varying PLQYs of 0.22, 0.02, and 0.12 for **54a**, **54b**, and **54c**, respectively, and notably retain fluorescence in the solid state. The |*g*_abs_| in the visible region increase systematically with helical length, reaching values as high as ≈10^−2^ for compounds **54b** and **54c –** among the highest reported to date – highlighting their strong potential in chiral optoelectronic applications (Table 18). In 2023, the same group reported a stable push–pull [7]helicene diimide (compound **55**) that exhibited notable chiroptical performance, with |*g*_abs_| and |g_lum_| values of 1.12 × 10^−2^ and 5.0 × 10^−3^, respectively, in toluene [70]. Furthermore, compound **55** demonstrated solvent-dependent fluorescence and CPL behavior across the visible spectrum, with both emission intensity and chiroptical properties varying in response to solvent polarity. Concurrently, Würthner’s group developed two naphthalimide-annulated [*n*]helicenes, compounds **56a** and **56b** (*n* = 5, 6), via a concise two-step synthetic route that afforded excellent yields and notable photophysical properties [71]. Both helicenes display high Φ_F_ as 73% for **56a** and 69% for **56b**. Notably, compound **56b** exhibits markedly enhanced |*g*_abs_| and |*g*_lum_| values of 2.1 × 10^−3^ and 2.3 × 10^−3^, approximately 4.5-fold greater than that of compound **56a**. Its red CPL emission at 615 nm and high *B*_CPL_ of 66.5 M^−1^ cm^−1^ underscore its potential for advanced chiral photonic applications.

**Table 18:** Structures and optical properties of **54a**–**c**, **55** and **56a**,**b**.

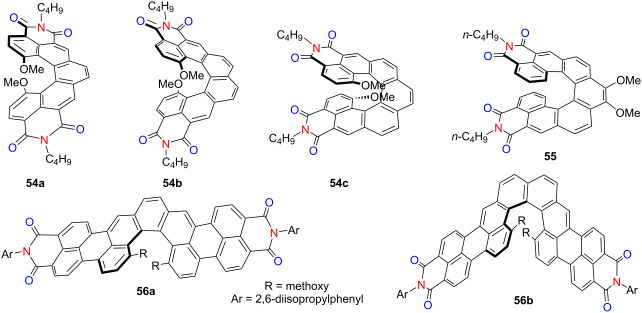						

compound	λ_abs(max)_ [nm]	λ_em(max)_ [nm]	Ф_F_	*|g*_abs_ *|*	|*g*_lum_|	*B*_CPL_ [M^−1^ cm^−1^]

**54a**	417, 442	471, 499	0.22^a^ 0.17^b^	7 × 10^−3^	–	–
**54b**	395	470, 498	0.02^a^ 0.02^b^	1.75 ×10^−2^	–	–
**54c**	452	508	0.12^a^ 0.06^b^	1.22 × 10^−2^	–	–
**55** ^c^	408	532	0.26	8.6 × 10^−3^	4.2 × 10^−3^	7.8
**56a**	629	655	0.73	4.5 × 10^−4^	5.0 × 10^−4^	22.0
**56b**	588	613	0.69	2.1 × 10^−3^	2.3 × 10^−3^	66.5

device	λ_EL(max)_ [nm]	*|g*_EL_ *|*	FWHM [nm]	CIE coordinate		EQE_max_ [%]

**56b**	618	–	50	–		2.3

^a^As detected in solution; ^b^as detected in the solid state; ^c^all detected in DCM.

Heteroatom engineering in double helicenes has emerged as a powerful strategy for tuning chiroptical properties and excited-state dynamics. In 2021, Sakamaki’s group synthesized a novel double N,O-hetero[5]helicene (compound **57b**) by coupling two 12*H*-benzo[*b*]phenoxazine (BPO) units and systematically compared it to its *N*,*N*-analogue (compound **57a**) derived from 13*H*-dibenzo[*b*,*i*]phenoxazine (DBPO) scaffolds [72] (Table 19). Compound **57b** was obtained in significantly higher yield and, like compound **57a**, exhibited electron-rich character and compact molecular packing, both favorable for *p*-type transistor performance. Importantly, both helicenes displayed strong CPL in CH_2_Cl_2_, with |*g*_lum_| values exceeding 10^−2^. Intriguingly, the CPL signals of the two compounds exhibited opposite signs, underscoring the sensitivity of chiral excited-state properties to heteroatom substitution within the helicene framework. Extending this design principle, the group reported a double *N*,*S*-hetero[5]helicene **58** constructed from two benzo[*b*]phenothiazine units in 2023 [73]. Compared to the *N*,*O*-analogue **57b**, this new compound showed more intense phosphorescence and an extended emission lifetime in dilute solution. Notably, it demonstrated room-temperature dual-emission CPL originating from both prompt fluorescence and long-lived phosphorescence, a rare feature in helicene systems. In a subsequent study, the same group reported a bis(*N*,*Se*)-hetero[4]helicene **59b** and systematically compared its structural and dynamic properties with those of its sulfur analogue **59a** [74]. Despite their close structural resemblance, the longer C–Se bond in **59b** led to a markedly higher racemization barrier (145.7 vs 112.8 kJ/mol), thereby illustrating how subtle atomic substitutions can significantly influence the conformational stability of helical molecules (Table 19). These studies illustrate how precise heteroatom modulation enables fine control over CPL directionality and emission lifetimes, offering promising avenues for the development of multifunctional chiral optoelectronic materials – particularly those capable of simultaneous fluorescence and phosphorescence-based CPL.

**Table 19:** Structures and optical properties of **57a**,**b**, **58**, and **59a**,**b**.

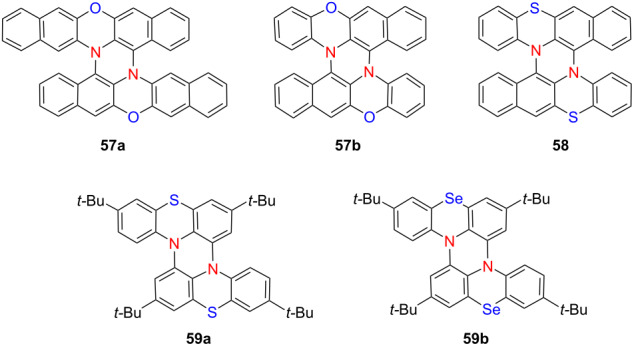					

compound	λ_abs(max)_ [nm]	λ_em(max)_ [nm]	Ф_F_	*|g*_abs_ *|*	|*g*_lum_|

**57a**	≈410	569	0.038	1.7 × 10^−2^	2.3 × 10^−2^
**57b**	≈380	587	0.035	1.3 × 10^−2^	1.3 × 10^−2^
**58**	≈390	547	0.003 0.30^a^	2.0 × 10^−2^	1.7 × 10^−2 b^
**59a**	380	–	–	–	–
**59b**	380	–	–	–	–

^a^Phosphorescence quantum yield Ф_P_; ^b^doped in β-estradiol matrix.

Recently, thiadiazole-fused helicenes have gradually come into our view. In 2023, Hirose’s group synthesized a series of tetraazadithia[*n*]helicenes – **60a**, **60b**, and **60c** – featuring 2,1,3-thiadiazole termini [75] (Table 20). Among them, compound **60c** exhibited pronounced CPL activity in toluene (|*g*_lum_| = 0.04, Φ_F_ = 3%), demonstrating the efficacy of terminal heterocycle incorporation for boosting chiroptical performance. In 2024, Babu and co-workers developed two π-extended hetero[6]helicenes – **61a** and **61b** – incorporating thiadiazole and selenadiazole moieties, respectively [76]. Substitution of sulfur with selenium enhanced intermolecular interactions and led to a notable reduction in the optical bandgap, highlighting the effectiveness of heteroatom modulation in tuning the electronic and photophysical properties of chiral nanographenes. These studies exemplify how strategic structural and electronic design – through π-extension, end-group heteroatom engineering, and atom-specific substitutions – enables precise tuning of chiroptical and photophysical properties in helicene-based materials, advancing their applicability in next-generation optoelectronic devices.

**Table 20:** Structures and optical properties of **60a**–**c** and **61a**,**b**.

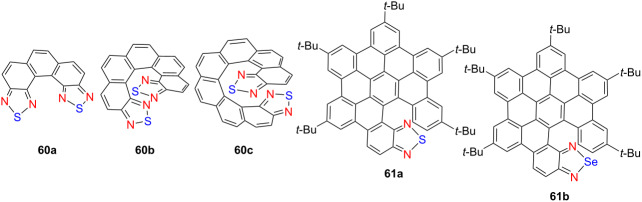						

compound	λ_abs(max)_ [nm]	λ_em(max)_ [nm]	Ф_F_	*|g*_abs_ *|*	|*g*_lum_|	*B*_CPL_ [M^−1^ cm^−1^]

**60a**	391	398	0.005	–	–	–
**60b**	431	450	0.008	1.5 × 10^−2^	1.0 × 10^−2^	2
**60c**	445	483	0.027	3.7 × 10^−2^	4.0 × 10^−2^	15
**61a**	340	536	0.0735	–	–	–
**61b**	349	556	0.009	–	–	–

In 2020, Pittelkow’s group developed a unique synthetic strategy that converts a non-planar hetero[7]helicene into a planar hetero[8]circulene featuring an antiaromatic cyclooctatetraene (COT) core (**62a**–**f**) [77] (Table 21). Through controlled oxidation of the thiophene units to sulfones, they achieved a systematic red-shift in both absorption and emission spectra. Remarkably, the emission of these derivatives spans nearly the entire visible spectrum. These studies provide innovative molecular design strategies for constructing helically twisted or planarized chiral π-conjugated systems with tunable optical properties, thereby paving the way for the development of multifunctional materials in advanced photonic and electronic technologies.

**Table 21:** Structure and optical properties of **62a**–**f**.

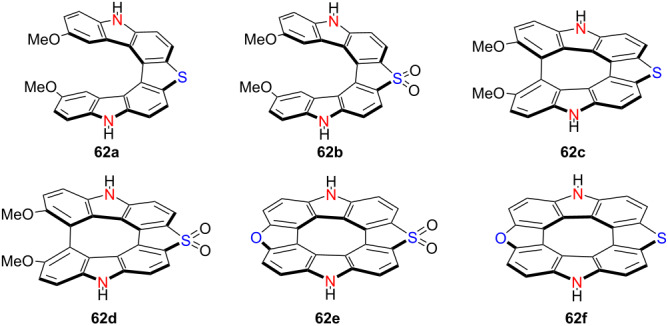					

compound	λ_abs(max)_ [nm]	λ_em(max)_ [nm]	Ф_F_	*|g*_abs_ *|*	|*g*_lum_|

**62a**	388	429	0.08	–	–
**62b**	419	484	0.25	–	–
**62c**	431	518	0.14	–	–
**62d**	476	574	0.13	–	–
**62e**	414	436	0.06	–	–
**62f**	473	485	0.12	–	–

In 2021, Viglianisi’s group synthesized a series of thia-bridged triarylamine[4]helicene-functionalized polynorbornenes **63a**–**c** via ring-opening metathesis polymerization (ROMP), introducing helicene chirality into polymer backbones with tunable electrochromic behavior [78]. These polymers exhibit reversible pH-responsive color changes. For instance, **63a** transitions from pale yellow to deep blue in the solid state upon exposure to TFA, while **63b** and **63c** in CH_2_Cl_2_ exhibit new absorption bands at 570 and 575 nm, respectively – reversibly decolorized upon triethylamine treatment (Table 22). This work demonstrates the potential of helicene-containing polymers as stimuli-responsive chiral electrochromic materials. In the same year, You’s group developed a transition-metal-catalyzed C–H/C–H-type regioselective C3-arylation of benzothiophenes using molecular oxygen as the oxidant [79]. This strategy afforded the TADF-active compound **64a**, which exhibits efficient blue emission and excellent OLED performance with a maximum EQE of 25.4%. This example highlights the utility of helicene-related heteroaromatic frameworks in the design of high-efficiency emissive materials. Also in 2021, Ema’s group reported a concise Scholl-type cyclodehydrogenation strategy for synthesizing azahelicenes and diaza[8]circulenes **65a**–**d** [24] (Table 22). These molecules exhibited distinct Cotton effects and CPL, with |*g*_lum_| reaching up to 1.6 × 10^−3^. This approach offers a generalizable route to structurally diverse chiral polycyclic aromatic hydrocarbons (PAHs) with strong chiroptical responses. Concurrently, Tanaka’s group achieved the enantioselective synthesis of aza[6]- and aza[7]helicene-like molecules via Rh(I)/chiral bisphosphine-catalyzed [2 + 2 + 2] cycloaddition [80]. The resulting S-shaped double aza[6]helicene-like compound **66** displayed high enantiomeric excess (up to 89% ee), pronounced chiroptical activity (|*g*_abs_| = 0.0054–0.0056), and substantial Φ_F_ of 0.21–0.32 under both neutral and acidic conditions. This work exemplifies the power of transition-metal catalysis for constructing enantioenriched helicenes with tunable photophysical properties. These contributions from 2021 underscore the synthetic versatility and functional diversity of helicene-based systems, spanning electrochromism, thermally activated delayed fluorescence, and circularly polarized luminescence. Such structural innovations provide valuable frameworks for the development of next-generation chiral optoelectronic materials.

**Table 22:** Structures and optical properties of **63a**–**c**, **64a**,**b**, **65a**–**d**, and **66**.

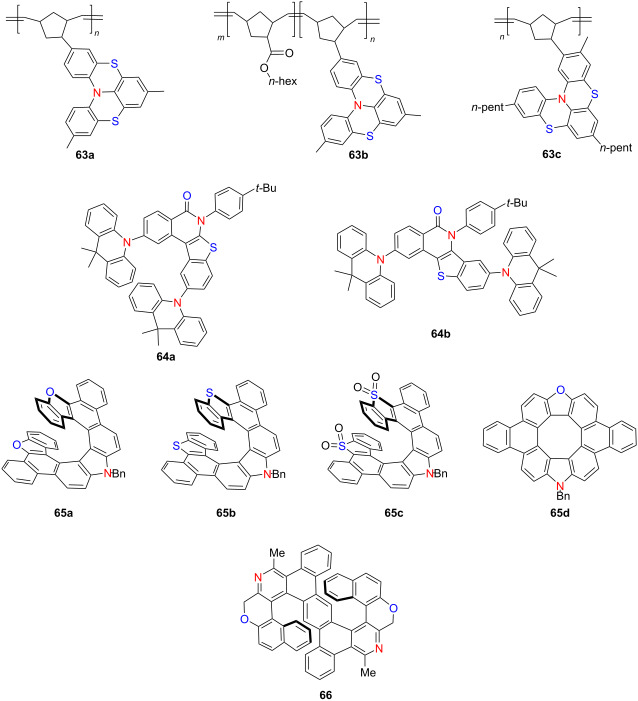						

compound		λ_abs(max)_ [nm]	λ_em(max)_ [nm]	Ф_F_	*|g*_abs_ *|*	|*g*_lum_|

**63a**		–	–	–	–	–
**63b**		570	–	–	–	–
**63c**		575	–	–	–	–
**64a**		376	456	–	–	–
**64b**		360	456	–	–	–
**65a**		401	420, 441	0.30	9.2 × 10^−4^	7.2 × 10^−4^
**65b**		414	432, 457	0.08	1.6 × 10^−3^	1.1 × 10^−3^
**65c**		440	493	0.10	7.3 × 10^−4^	2.6 × 10^−4^
**65d**		420	554	0.02	–	–
**66**		388, 431	489	0.21	5.59 × 10^−3^	1.42 × 10^−3^
**66** (+TFA)		290, 389, 439	555	0.32	4.98 × 10^−3^	1.38 × 10^−3^

device	λ_EL(max)_ [nm]	|*g*_EL_|	FWHM [nm]	CIE coordinate		EQE_max_ [%]

**64a**	474	–	–	(0.15, 0.23)		25.4

In 2022, Furuta’s group developed a one-pot synthetic protocol to access (NH)-phenanthridinone derivatives and chiral amide-functionalized [7]helicene-like molecules **67a**,**b** from biaryl dicarboxylic acids, employing a Curtius rearrangement followed by basic hydrolysis [81] (Table 23). Notably, when chalcogen-containing substrates were used, the process afforded phosphorus ester derivatives of aza[5]helicenes. The chiral nature of the products was confirmed by optical rotation and CD measurements. In parallel, Soni’s group established an efficient three-step synthesis of coumarin-containing hetero[5]- and [6]helicene-like structures **68a**–**g** in high yields [82]. These compounds display diverse photophysical behaviors: compound **68d** emits yellow fluorescence in both solution and solid state, exhibiting solvatofluorochromism due to a twisted intramolecular charge transfer (TICT) mechanism, while compound **68e** emits blue light (Φ_F_ = 0.37) and demonstrates pronounced AIE in the solid state. Concurrently, Jiang’s group reported **69b**, the first hetero[4]helicene-type molecule exhibiting both CPL and TADF [83]. This compound displays a high Φ_F_ of 0.51 and a |*g*_lum_| of 1.2 × 10^−3^. OLED devices fabricated using **69b** emit sky-blue light with a peak EQE of 10.6% and |*g*_EL_| values up to 1.6 × 10^−3^. Collectively, these studies demonstrate the versatility of helicene-inspired architectures for constructing multifunctional chiral optoelectronic materials, highlighting their growing relevance in next-generation circularly polarized OLED technologies.

**Table 23:** Structures and optical properties of **67a,b**, **68a**–**g**, and **69a**,**b**.

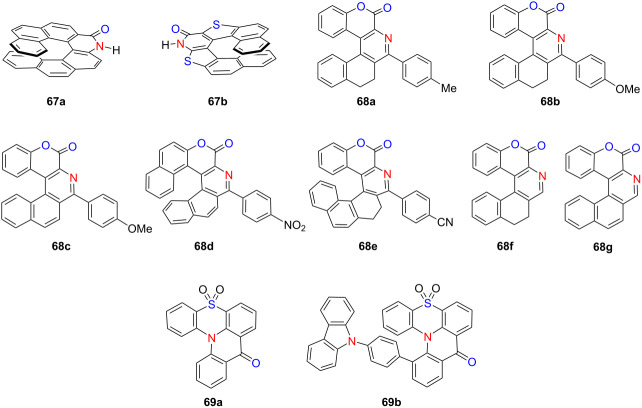						

compound		λ_abs(max)_ [nm]	λ_em(max)_ [nm]	Ф_F_	*|g*_abs_ *|*	|*g*_lum_|

**67a**		–	–	–	–	–
**67b**		–	–	–	–	–
**68a**		295	411	0.08	–	–
**68b**		309	422	0.10	–	–
**68c**		328	439	0.03	–	–
**68d**		394	514	0.22	–	–
**68e**		320	423	0.37	–	–
**68f**		318	389	0.01	–	–
**68g**		317	411	0.04	–	–
**69a**		397	431	–	–	–
**69b**		400	446	0.51	–	1.2 × 10^−3^

device	λ_EL(max)_ [nm]	|*g*_EL_|	FWHM [nm]	CIE coordinate		EQE_max_ [%]

**69b**	488	1.6 ×10^−3^	72	(0.17, 0.34)		10.6

Takizawa and co-workers have pioneered electrochemical strategies for synthesizing structurally diverse hetero[7]helicenes with tunable chiroptical properties and excellent configurational stability. In 2022, they introduced two electrochemical routes to construct aza-oxa-dehydro[7]helicenes, yielding helicenes with high racemization barriers and notable chiral stability [84]. The quasicirculenes **70a** and **70b** demonstrated strong blue CPL activity, with |*g*_lum_| values of 2.5 × 10^−3^ at 433 nm and 2.4 × 10^−3^ at 418 nm, respectively (Table 24). Building on this, the team achieved the enantioselective synthesis of heterodehydrospiroenes on a gram scale using chiral vanadium(V) complexes – marking a significant advancement in asymmetric electrochemical catalysis. In a complementary study that same year, they reported a two-step electrochemical synthesis of a double aza-oxa[7]helicene via oxidative coupling followed by dehydrative cyclization [85]. The resulting meso-isomer (*P*,*M*)**-71** emerged as the major product, exhibiting dual emission bands at 415 and 440 nm and solvent-independent absorption at 407 nm. Expanding the structural diversity, the group developed a two-pot synthesis of unsymmetrical hetero[7]helicenes **72a**–**g** in 2023 [86], employing *p*-benzoquinone and *N*-aryl-2-naphthylamines through acid-promoted cyclization followed by electrochemical domino reactions. This method produced six compounds with yields ranging from 33–45%, all featuring extended π-conjugation and distinct photophysical characteristics. Furthermore, they established a mild electrochemical protocol for synthesizing oxaza[7]helicenes incorporating pyrrole and furan units [87]. This method afforded products in 50–86% yield with Faradaic efficiencies up to 77%. Among them, derivative **73** exhibited CPL activity (|*g*_lum_| = 3.0 × 10^−4^), showcasing the ability to modulate chiroptical responses via heteroatom integration. These studies underscore the versatility of electrochemical synthesis in enabling precise structural modulation of heterohelicenes, facilitating access to high-performance chiral optoelectronic materials.

**Table 24:** Structures and optical properties of **70a**,**b**, **71**, **72a**–**g**, and **73**.

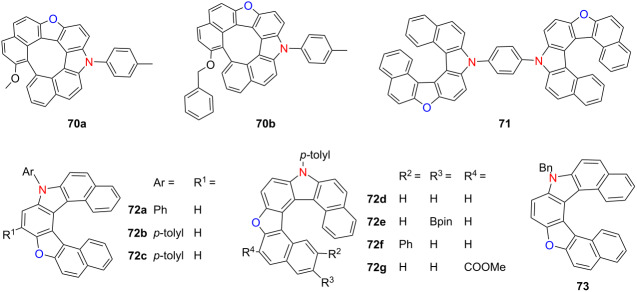					

compound	λ_abs(max)_ [nm]	λ_em(max)_ [nm]	Ф_F_	*|g*_abs_ *|*	|*g*_lum_|

**70a**	402	433	0.25	–	2.5 × 10^−3^
**70b**	–	418	0.16	–	2.4 × 10^−3^
**71**	407	415, 440	–	–	–
**72a**	406	439	–	–	–
**72b**	403	440	0.065	–	–
**72c**	402	440	–	–	–
**72d**	413	450	–	–	–
**72e**	401	440	–	–	–
**72g**	405	440	–	–	–
**73**	–	–	–	–	3.0 × 10^−4^

In 2023, Zhang’s group introduced a new class of helically chiral double hetero[4]helicenes **74a** and **74b** exhibiting CP-TADF, constructed on a distinct donor–acceptor core architecture [88] (Table 25). These compounds demonstrate excellent configurational stability and robust CPL signals both in solution and in solid-state films, with a |*g*_lum_| of 3.1 × 10^−3^. Corresponding CP-OLEDs based on compound **74a** achieved outstanding device performance, reaching a maximum EQE of 20.03% and a |*g*_EL_| of 2.9 × 10^−3^ – underscoring their considerable potential for advanced chiral optoelectronic applications. Building upon this framework, in 2024, the same group developed a novel cove-region bridging strategy to construct double hetero[4]helicenes with enhanced structural rigidity and persistent chirality [89]. By selectively modifying the bay regions of the SPZ (spiro[fluorene-9,9'-xanthene]) scaffold, they successfully converted initially non-emissive helicenes into efficient TADF luminophores with tunable emission wavelengths ranging from sky-blue to deep red. Particularly, the enantiomeric forms of the **75b** derivatives emerged as rare examples of red-emissive CPL materials. This innovative design approach offers a versatile and modular platform for engineering chiral multi-helicene systems with customizable optoelectronic properties, paving the way for their deployment in next-generation CPL-active materials and high-performance CP-OLED devices.

**Table 25:** Structures and optical properties of **74a**,**b** and **75a**–**c**.

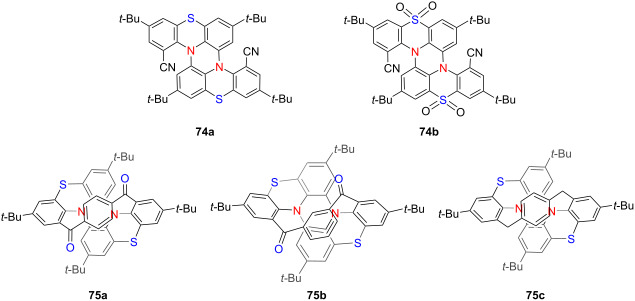					

compound	λ_abs(max)_ [nm]	λ_em(max)_ [nm]	Ф_F_	*|g*_abs_ *|*	|*g*_lum_|

**74a**	406	493	0.13/0.67^a^	–	3.1 × 10^−3,a^
**74b**	357	450	0.07/0.22^a^	–	–
**75a**	612	–	–	–	–
**75b**	495	656	0.02	–	2.7 × 10^−3^
**75c**	436	480	0.09	–	2.5 × 10^−2^

device	λ_EL(max)_ [nm]	|*g*_EL_|	FWHM [nm]	CIE coordinate	EQE_max_ [%]

(*M,M*)-**74a**	500	2.9 ×10^−3^	82	(0.24, 0.50)	20.03
rac-**74a**	500	–	81	(0.24, 0.49)	20.00

^a^Detected as 20 wt % doped films with the mCBP host.

In 2024, Jančařík and co-workers introduced an intramolecular radical cyclization strategy to synthesize highly luminescent tetraceno[6]helicenone and its aza analogue **76** [90] (Table 26). The incorporation of a carbonyl group into the helicene backbone substantially enhanced fluorescence quantum yields and red-shifted the emission into the visible region. The aza analogue demonstrated promising performance in OLEDs, confirming its potential for optoelectronic applications. Concurrently, Shirinian’s group synthesized a series of nitrogen-functionalized quinoline (NFQ)-based aza-oxa[5]helicenes **77a**–**f** exhibiting excellent UV stability and solvent-dependent fluorescence [91]. Protonation significantly enhanced their emission intensity, and the presence of nitrogen facilitated further structural derivatization. In the same year, Alcarazo’s group reported an enantioselective gold-catalyzed synthesis of compound **78**, achieving a high enantiomeric excess [92]. They further investigated various post-synthetic modification strategies, demonstrating their potential for application in chiral photonic materials. Collectively, these advances underscore the power of structural tailoring, heteroatom incorporation, and enantioselective strategies in finely tuning the photophysical and chiroptical properties of helicenes, providing a versatile foundation for the development of high-performance chiral optoelectronic materials.

**Table 26:** Structures and optical properties of **76**, **77a**–**f**, and **78**.^a^

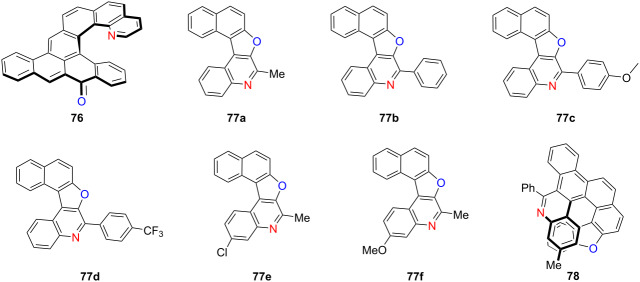					

compound			λ_abs(max)_ [nm]	λ_em(max)_ [nm]	Ф_F_

**76**			483	561	0.43
**77a** in CHCl_3_			352	379, 399	0.39
**77b** in CHCl_3_			359	379, 392	0.04
**77c** in CHCl_3_			360	397	0.08
**77d** in CHCl_3_			362	390, 403	0.09
**77a** in heptane			347	388	0.21
**77e** in heptane			348	391	0.20
**77f** in heptane			348, 358	383	0.19
**77a** in toluene			352	394, 421	0.56
**77e** in toluene			353	380, 400	0.44
**77f** in toluene			353	388	0.28
**77a** in acetonitrile			348	375	0.48
**77e** in acetonitrile			348	383	0.48
**77f** in acetonitrile			349	391	0.42
**77a** in methanol			351	383	0.48
**77e** in methanol			349	391	0.47
**77f** in methanol			352	396	0.27

device	λ_EL(max)_ [nm]	|*g*_EL_|	FWHM [nm]	CIE coordinate	EQE_max_ [%]

**76**	580	–	103	–	0.15
**76**:MADN 95:5	550	–	93	–	0.7

^a^No *g*_abs_ or *g*_lum_ values were reported, no optical characterization for **78**.

## Conclusion

Nitrogen-doped helicenes and their heteroatom co-doped analogues constitute a rapidly advancing class of chiral π-conjugated materials, distinguished by exceptional structural tunability, photophysical diversity, and chiroptical functionality. The integration of nitrogen – and its synergistic pairing with heteroatoms such as boron, oxygen, sulfur, and selenium – has significantly expanded the molecular design space, enabling precise control over redox behavior, emission wavelength, CPL, and responsiveness to thermal or redox stimuli. These heteroatom modifications have led to remarkable breakthroughs, including near-unity PLQYs, ultranarrow emission bands, |*g*_lum_| values exceeding 10^−3^, and unprecedented *B*_CPL_, particularly in the visible to near-infrared (NIR) spectral regions.

Recent advances in synthetic methodology – including electrochemical, Scholl-type, and enantioselective catalytic strategies – have further enabled access to structurally complex helicene topologies with enhanced configurational stability and integrated multifunctionality. These developments have facilitated a growing range of applications in CP-OLEDs, molecular sensing, chiral switches, and photonic devices. Moving forward, key challenges remain, such as mitigating spectral broadening in red/NIR emission, enhancing the chemical and photostability of electron-deficient helicenes, and developing sustainable, scalable synthetic approaches. The integration of computational design with multifunctional molecular engineering is expected to accelerate the deployment of helicene-based materials in next-generation technologies spanning chiral optoelectronics, bioimaging, spintronics, and quantum information science.

## Data Availability

Data sharing is not applicable as no new data was generated or analyzed in this study.
